# Honokiol Mitigates Metabolic-Associated Fatty Liver Disease by Regulating Nrf2 and RIPK3 Signaling Pathways

**DOI:** 10.5152/tjg.2024.23470

**Published:** 2024-07-01

**Authors:** Wen Cao, Zengdian Chen, Chenhui Lin, Xiaojin Lin, Yang Chen, Jingjuan Zhang

**Affiliations:** Department of Gastroenterology, Fuzhou Second General Hospital, Fuzhou, Fujian Province, China

**Keywords:** Honokiol, metabolic-associated fatty liver disease, Nrf2 signaling pathways, RIPK3

## Abstract

**Background/Aims:**

Metabolic-associated fatty liver disease (MAFLD) is a common cause of chronic liver disease worldwide. However, there is currently no recognized effective drugs for treating it.

**Materials and Methods:**

In this study, we investigated the efficacy of Honokiol (HNK) in vitro for mitigating MAFLD. Then, 0.4 mM palmitic acid (PA) and LO2 cells were used to establish the MAFLD model. The protective effect of HNK on MAFLD was confirmed by Oil Red O staining and cell counting kit (CCK-8) assay in LO2 cell line. Quantitative real-time polymerase chain reaction (qRT-PCR) and Western blot were carried out to analyze the regulatory role of HNK on Nrf2 and RIPK3 signaling pathways. The effect of HNK and its downstream signaling pathways on oxidative stress were verified by the detection of reactive oxygen species (ROS), malondialdehyde (MDA), catalase (CAT), and superoxide dismutase (SOD). The concentration of IL-1β, IL-6L, and TNF-α was assessed by enzyme-linked immunosorbent assay (ELISA).

**Results:**

The middle concentration of HNK (50 μmol/L) was selected as the best option for inhibiting lipidosis and oxidative stress in MAFLD models. Honokiol mitigates MAFLD via activation of nuclear factor E2-related factor 2 (Nrf2) signaling pathways in vitro. Honokiol suppressed MAFLD via activating the Nrf2 signaling pathway to play an antioxidant and anti-inflammatory role. Also, HNK regulates Nrf2 and RIPK3 signaling pathways to mitigate MAFLD.

**Conclusion:**

Our results showed that HNK may suppress the oxidative stress and inflammation in MAFLD via activation of Nrf2 signaling pathway.

**Main Points**

- The lipidosis and oxidative stress in the metabolic-associated fatty liver disease (MAFLD) model are suppressed by the middle concentration of Honokiol (HNK).
- HNK mitigates MAFLD via activation of the Nrf2 signaling pathways in vitro.
- HNK suppressed the MAFLD by antioxidation and anti-inflammation effect via the activation of the Nrf2 signaling pathway.

## Introduction

Metabolic-associated fatty liver disease (MAFLD) is a common chronic liver disease worldwide that causes excessive deposition of triglycerides in the liver due to genetic susceptibility, overnutrition, and its complications.^1,2^ The MAFLD disease spectrum includes nonalcoholic fatty liver (NAFL), nonalcoholic steatohepatitis (NASH), and hepatocellular carcinoma (HCC).^3,4^ The incidence rate of MAFLD has increased year by year.^5^ In Türkiye, a MAFLD-related cohort study showed a high prevalence of hepatic steatosis (60.1%) while the prevalence of gallbladder stones was 7.6% among the participants.^6^ Therefore, the exploration of effective intervention measures is crucial. However, there is currently no recognized effective drug for the treatment of MAFLD. At present, the recognized effective treatment for MAFLD is still to reduce the weight of patients through lifestyle intervention.^7^ This project aims to conduct in-depth research on the relevant mechanisms of MAFLD and search for effective therapeutic drugs, which are of great significance for alleviating the condition of MAFLD patients, improving their survival rate, and quality of life.

Mard et al have reported that crocin can be introduced as a candidate hepatoprotective agent against NASH by virtue of its anti-inflammatory, antioxidant, and antiapoptotic properties.^8^ Herein, we investigated the effect of another compound, Honokiol (HNK), on MAFLD. Honokiol is a lignan compound extracted from the traditional Chinese medicine *Magnolia officinalis*,^9,10^ which has positive effects such as anti-inflammatory,^11^ antioxidant,^12^ and improving glucose and lipid metabolism.^13^ In recent years, HNK has been proven to intervene in the progression of various liver diseases. For instance, Kataoka et al have shown that HNK can inhibit TGF-β1/SMAD signaling in hepatic stellate cells.^13,14^ Lee et al^15^ also confirmed that HNK can mitigate HCC cell migration through downregulating Cyclophilin B expression. The above studies suggested that HNK has potential therapeutic effects on MAFLD, while the specific mechanism still needs further in-depth research.

Oxidative stress is widely recognized to be a vital biochemical process in the occurrence and development of MAFLD.^16^ Nuclear factor E2-related factor 2 (Nrf2) is an important endogenous antioxidant stress pathway and has been reported to play an important role in MAFLD. Afonso et al^17^ found that the expression level of receptor-interacting serine-threonine kinase 3 (RIPK3) increased in MAFLD patients. In addition, a previous research indicated that intermittent hypoxia can exacerbate MAFLD by promoting RIPK3-dependent necroptosis and inhibiting the Nrf2 signaling pathway.^18^ Hence, the effect of HNK on Nrf2 and RIPK3 signaling was investigated in this study.

## Materials and Methods

### Cell Culture and Grouping

LO2 cells were cultured in RPMI 1640 complete medium (containing 10% FBS, 100 U/mL penicillin, 100 μg/mL streptomycin) in the incubator (37°C, 5% CO2, saturated humidity). The palmitic acid (PA) (Sigma, P5585), HNK (Meilunbio, MB5989), Nrf2 inhibitor (APExBIO, B8300), and RIPK3 inhibitor (APExBIO, B8589) were used to treat the LO2 cells. Then, the cells were treated with 0.4 mM PA for 72 hours to construct an MAFLD model.^19^ Also, different concentrations of HNK, as well as Nrf2 inhibitor and RIPK3 inhibitor, were used to treat the cells to explore the potential regulatory mechanisms of HNK in the text.

The grouping and treatments for cells are as follows:

- Control group: LO2 cells.
- MAFLD group: LO2 cells + 0.4 mM palmitic acid (PA) for 72 hours.
- HNK (high/middle/low dose; 25/50/100 μmol/L) group: LO2 cells + 0.4 mM PA + HNK (100/50/25 μmol/L) for 48 hours.
- HNK + Nrf2 inhibitor group: LO2 cells + 0.4 mM PA + HNK (50 μmol/L) + Nrf2 inhibitor (2 μmol/L) for 48 hours.
- RIPK3 inhibitor group: LO2 cells + 0.4 mM PA + RIPK3 inhibitor (0.1 μmol/L) for 48 hours.
- Nrf2 inhibitor + RIPK3 inhibitor group: LO2 cells + 0.4 mM PA + Nrf2 inhibitor (2 μmol/L) + RIPK3 inhibitor (0.1 μmol/L) for 48 hours.

### Oil Red O Staining

LO2 cells were cultured in a 24-well plate at a density of 5 × 10^6^ cells/mL. When the cell confluency reaches about 60%, an appropriate amount of 4% paraformaldehyde was added to each well to fix the cells for 15 minutes. After being washed with distilled water, each well was immersed in 60% isopropanol for 20-30 seconds, and then the isopropanol was added. Each well was stained with 500 μL of oil red O staining solution for 15 minutes, followed by washing with isopropanol to remove the dye solution. Then, each well was stained with a hematoxylin staining solution for 2 minutes and washed with distilled water. Finally, the samples were dried and sealed.

### Cell Counting Kit (CCK-8 Assay)

LO2 cells (8 × 10^4^ cells/mL) were cultured in a 96-well plate. Each group was equipped with eight composite wells, and they were cultured until LO2 cells adhered to the wall. Then, 10 μL of CCK-8 enhanced solution was added to each well and incubated for 1 h. The microplate reader was used to determine the absorbance of each sample at 450 nm.

### Quantitative Real-Time Polymerase Chain Reaction

Total RNA was isolated from LO2 cells by using TRIzol. Complementary DNA (cDNA) was synthesized from total RNA using a reverse transcription kit. Target gene expressions were detected using AceQ Universal SYBR qPCR Master Mix on the ABI 7500 PCR system. The cycling parameters used were 95°C for 15 seconds, 55-60°C for 15 seconds, and 72°C for 15 seconds for 45 cycles. Ct values were determined during the exponential amplification phase of real-time PCR. The GAPDH gene was used as the reference gene. The 2^−△△Ct^ method was used to calculate the relative expression levels of the genes.^20^ All experiments were performed in triplicate. The specific primer sequences were as follows:

TGF-β1 Forward: 5′-GGCCAGATCCTGTCCAAGC-3′;
TGF-β1 Reverse: 5′-GTGGGTTTCCACCATTAGCAC-3′;
TIMP1 Forward: 5′-CTTCTGCAATTCCGACCTCGT-3′;
TIMP1 Reverse: 5′- ACGCTGGTATAAGGTGGTCTG-3′;
Collagen 1 Forward: 5′-GAGGGCCAAGACGAAGACATC-3′;
Collagen 1 Reverse: 5′-CAGATCACGTCATCGCACAAC-3′;
GAPDH Forward: 5′-ATGGGGAAGGTGAAGGTCG-3′;
GAPDH Reverse: 5′-TCGGGGTCATTGATGGCAACAATA-3′.

### Detection of Pro-inflammatory Cytokines

The supernatant of cells in each group was collected. The concentrations of interleukin 1β (IL-1β) (Jianglaibio, JL18442), interleukin 6 (IL-6) (Jianglaibio, JL20269), and tumor necrosis factor α (TNF-α) (Jianglaibio, JL10484) in the supernatant were assessed based on the instructions of the ELISA kits. All ELISA experiments were performed in triplicate.

### Detection of Oxidative Factors

Serum-free culture medium was used to dilute 2′,7′-dichlorodihydrofluorescein diacetate (DCFH-DA), and LO2 cells were grouped and treated as previously described. The supernatant was discarded, and the LO2 cells were added to DCFH-DA and incubated at 37°C for 20 minutes. The suspension was then used for flow cytometry detection of reactive oxygen species (ROS) (Em 488 nm, Ex 525 nm). The oxidative stress level of the cell supernatant was detected based on the instructions of the malondialdehyde (MDA) (Nanjing Jiancheng Bioengineering, A003-2), superoxide dismutase (SOD) (Nanjing Jiancheng Bioengineering, A001-3), catalase (CAT) (Solarbio, BC0200) kits.

### Western Blot

Radioimmunoprecipitation assay (RIPA) lysis buffer was utilized to extract total protein from each group of LO2 cells. The protein was separated by SDS-PAGE and transferred to the PVDF membrane. The samples were added with the primary antibody and incubated overnight at 4°C. Then samples were added with HRP-labeled IgG antibodies and incubated at 25°C for 1 hour. The samples were processed by ECL and captured by the gel imaging system. The grayscale values of the target bands were determined using Image J software and normalized to GAPDH. The purchased antibodies are listed as follows: GAPDH (1:10 000, Proteintech, cat# 60004-I-Ig), TGF-β1 (1:4000, Proteintech, cat# 21898-1-AP), TIMP1 (1:2000, Boshide, cat# A00561), Collagen I (1:1000, Affinity, cat# AF7001), Nrf2 (1:1000, Abcam, cat# ab62352), HO-1 (1:3000, Proteintech, cat# 27282-1-AP), NQO1 (1:8000, Proteintech, cat# 11451-1-AP), RIP1 (1:3000, Proteintech, cat# 17519-1-AP), RIP3 (1:4000, Proteintech, cat# 17563-1-AP), MLKL (1:10 000, Proteintech, cat# 66675-1-lg), and RP-conjugated Affinipure Goat Anti-Rabbit IgG(H+L) (Proteintech, cat# SA00001-2). A Predyed Rainbow Protein marker (10-190KD) (cat. MA0342, Meilunbio) was used as the protein marker.

### Statistical Analysis

GraphPad Prism 8.0 (GraphPad Software, San Diego, California, USA) was utilized to conduct the statistical analyses. Data were summarized as mean ± SD from at least three independent experiments performed in triplicate. The student’s *t-*test was used to compare differences between the two groups. *P* < .05 was considered statistically significant.

## Results

### HNK Suppressed the Oxidative Stress in MAFLD In Vitro

**Figure 1.**
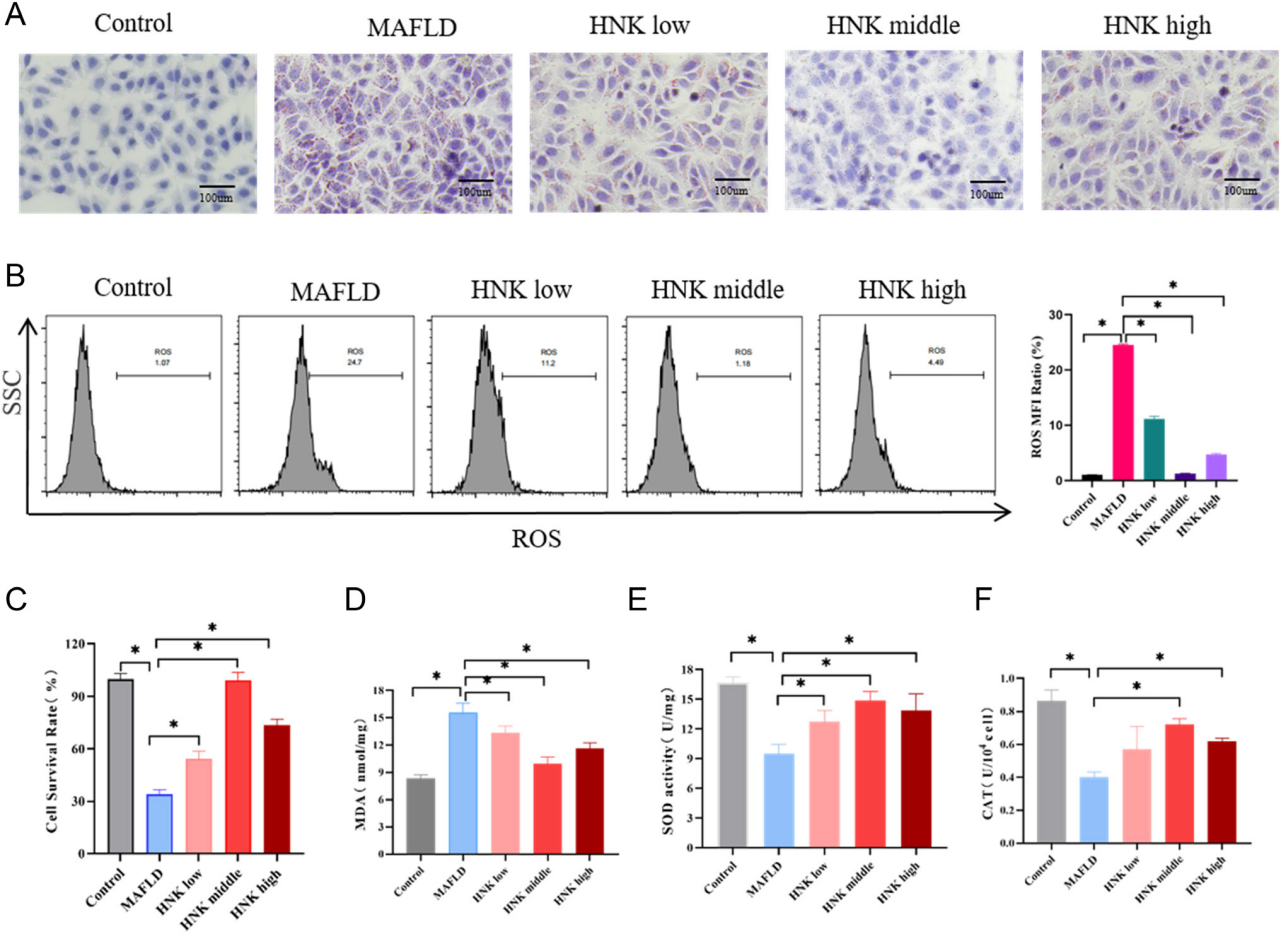
HNK suppressed the oxidative stress in MAFLD in vitro. (A) The oil red staining was used to evaluate the fat levels in LO2 cells, MAFLD model, MAFLD model treated with high, middle, and low concentration of Honokiol (HNK). Bar = 100 μm. (B) Flow cytometry was used to detect the ROS level in LO2 cells with different treatments. (C) The CCK-8 assay was used to measure the cell survival rate of LO2 cells with different treatments. (D-F) The concentrations of MDA (D) and activity of SOD (E) and CAT (F) were determined by the biochemical detection kits. N ≥ 3, *P < .05 was shown as significance between the 2 groups. Student’s *t*-test was used to compare differences between the 2 groups.

First, the antioxidative effect of HNK on MAFLD was investigated in the LO2 cell line. The MAFLD model was established based on the LO2 cell line via induction with 0.4 mM PA for 72 hours. The results of oil red O staining in the MAFLD model presented the most lipid droplet deposition in liver cells. With the treatment of HNK, the lipid droplets were reduced compared to the MAFLD group, especially in the middle dose of HNK (Figure 1A). In addition, we found that ROS levels were increased in MAFLD but significantly decreased in HNK treatment, especially in the middle dose group (*P* < .05) (Figure 1B). Furthermore, the cell survival rate was lowest in the MAFLD group but improved with HNK treatment, especially in the middle dose of HNK (*P* < .05) (Figure 1C). The concentration of MDA was increased in the MAFLD group, while it was reduced by HNK treatment (*P* < .05) (Figure 1D). The activities of SOD and CAT were reduced in the MAFLD group but enhanced by HNK treatment (*P* < .05) (Figure 1E and 1F). These aforementioned pieces of evidence indicated that lipidosis and oxidative stress in the MAFLD model were suppressed by HNK, especially in the middle concentration of HNK. Subsequently, the middle dose of HNK was selected for the following experiments.

### HNK Mitigated MAFLD Via Activation of Nrf2 Signaling Pathways In Vitro

**Figure 2.**
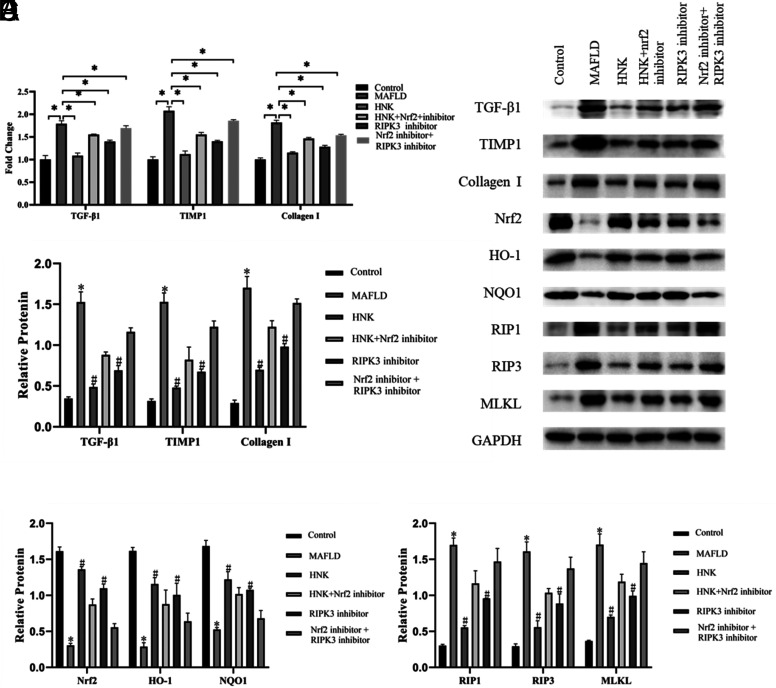
HNK regulated the Nrf2–RIPK3 signaling pathway. (A) The mRNA expression levels of fibrosis-related factors (TGF-β1, TIMP1, and collagen I) in the control group, MAFLD model group, HNK group, HNK+Nrf2 inhibitor group, RIPK3 inhibitor group, and Nrf2 inhibitor + RIPK3 inhibitor group were detected by qPCR. *P < .05 was shown as significance between the 2 groups. Student’s *t*-test was used to compare differences between the 2 groups. (B-E) The protein expression levels of fibrosis-related factors (TGF-β1, TIMP1, and collagen I), key factors in the Nrf2 signaling pathway (Nrf2, HO-1, and NQO1), and key factors RIPK3 signaling pathway in LO2 cells with different treatments were detected by western blot. n = 3, *P < .05, compared with the control group; ^#^ *P* < .05, compared with the MAFLD group. Student’s *t*-test was used to compare differences between the 2 groups.

In the next step, we sequentially investigated the underlying mechanism of HNK in the Nrf2 signaling pathway. The mRNA expression levels of fibrosis-related factors (TGF-β1, TIMP1, and collagen I) were detected (Figure 2A). In the MAFLD group, the mRNA expression levels of fibrosis-related factors were remarkably enhanced, while they were reduced by HNK treatment. With the treatment of an Nrf2 inhibitor, the mRNA expression levels of fibrosis-related factors were considerably increased compared to the HNK group (*P* < .05). The trend of protein levels of fibrosis-related factors (TGF-β1, TIMP1, and collagen I) was consistent with the mRNA levels (*P* < .05) (Figure 2B and 2C). These results suggested that HNK regulated Nrf2 signaling pathways to mitigate the fibrosis of LO2 cells.

The protein expression levels of Nrf2, HO-1, and NQOI were lessened in the MAFLD group but enhanced by the HNK treatment. With the treatment of the Nrf2 inhibitor, the protein expression levels of Nrf2, HO-1, and NQOI were significantly decreased compared with the HNK group, especially with the treatment of both (*P* < .05) (Figure 2B and 2D). These pieces of evidence indicate that HNK activates the Nrf2 signaling pathway in MAFLD.

**Figure 3.**
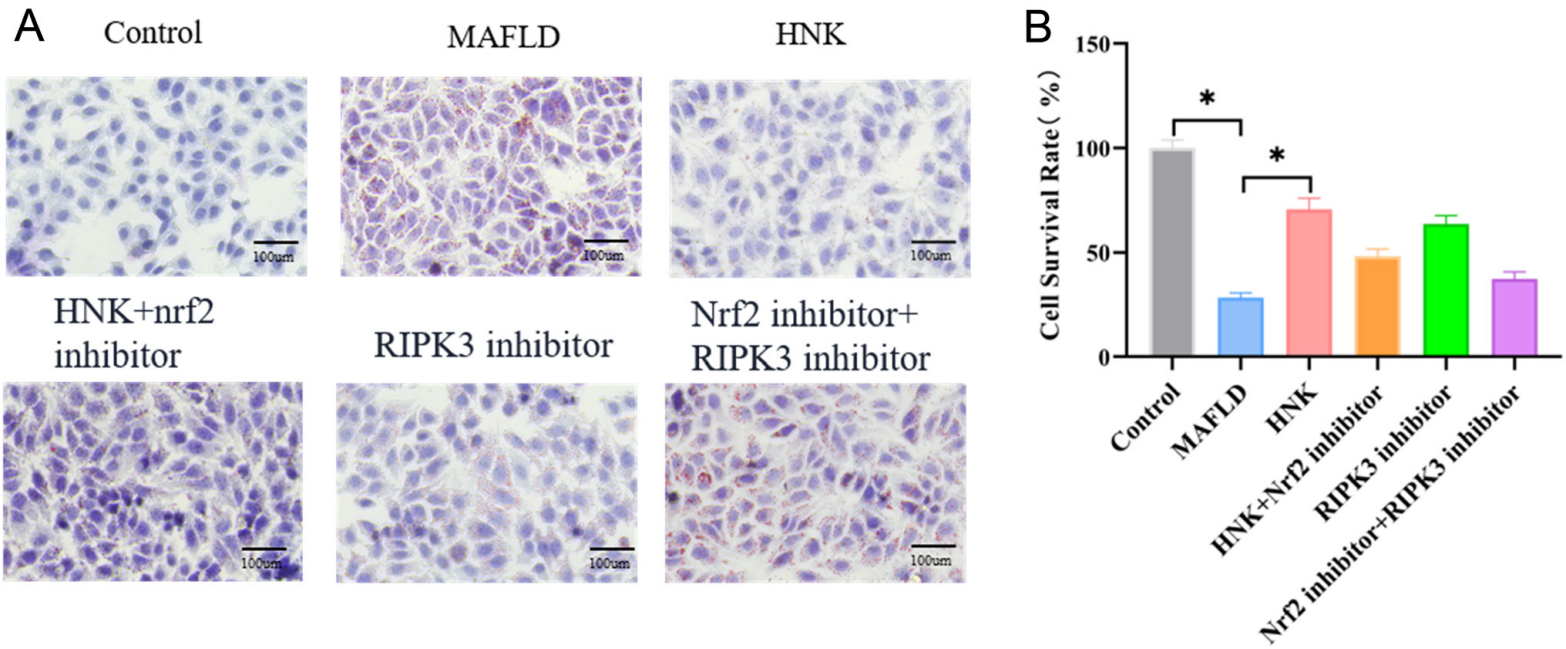
HNK inhibited MAFLD progression by activating the Nrf2 signaling pathway. (A) The oil red stain was utilized to evaluate the fat levels in LO2 cells, the MAFLD model, MAFLD model treated with HNK, RIPK3 inhibitor, Nrf2 inhibitor + RIPK3 inhibitor, respectively. Bar = 100 μm. (B) CCK-8 assay was used to measure the cell survival rate of LO2 cells with different treatments. n = 8, *P < .05 was shown as significance between the 2 groups. Student’s *t*-test was used to compare differences between the 2 groups.

After LO2 cells with different treatments were stained with oil red stain, the MAFLD group presented the most lipid droplet deposition, and the HNK group showed the least. With the treatment of the Nrf2 inhibitor, the lipid droplet was observed to be enhanced compared with the MAFLD group (*P* < .05) (Figure 3A). The cell survival rate of LO2 cells was lowest in the MAFLD group and highest in the HNK group, while the cell survival rate was lessened by the Nrf2 inhibitor (*P* < .05) (Figure 3B). The evidence above revealed that HNK induced the activation of the Nrf2 signaling pathway to mitigate MAFLD.

### The Negative Feedback Axis of Nrf2 Pathway and RIPK3/MLKL Necroptosis Signaling in MAFLD

Then, the underlying mechanism of Nrf2 in MAFLD was continually explored. As shown in Figure 2A-C, the mRNA and protein expression levels of fibrosis-related factors (TGF-β1, TIMP1, and collagen I) were remarkably decreased in the RIPK3 inhibitor group compared with the MAFLD group, while they were increased in the RIPK3 inhibitor + Nrf2 inhibitor group compared with the RIPK3 inhibitor group (*P* < 0.05).

The protein expression levels of Nrf2, HO-1, and NQOI were enhanced in the RIPK3 inhibitor group compared with the MAFLD group. Interestingly, these key proteins in the Nrf2 signaling pathway were decreased in the RIPK3 inhibitor + Nrf2 inhibitor group compared with the RIPK3 inhibitor group (*P* < .05) (Figure 2B and 2D). Furthermore, the protein expression levels of RIP1, RIP3, and MLKL were considerably decreased in the RIPK3 inhibitor group compared with the MAFLD group. Nevertheless, these key proteins in the RIPK3/MLKL necroptosis signaling pathway were decreased in the RIPK3 inhibitor + Nrf2 inhibitor group compared with the RIPK3 inhibitor group (*P* < .05) (Figure 2B and 2E).

With the treatment of the RIPK3 inhibitor, the lipid droplet was observed to be reduced in the RIPK3 inhibitor group compared to the MAFLD group, while it was increased compared to the RIPK3 inhibitor + Nrf2 inhibitor group (*P* < .05) (Figure 3A). Nevertheless, the cell survival rate of LO2 cells was increased in the RIPK3 inhibitor group compared to the MAFLD group, while it was decreased in the RIPK3 inhibitor group compared to the RIPK3 inhibitor + Nrf2 inhibitor group (*P* < .05) (Figure 3B). These results suggested the negative feedback axis of Nrf2 pathway and RIPK3/MLKL necroptosis signaling in MAFLD.

### HNK Suppressed the MAFLD by Antioxidation and Anti-inflammation Effect via Activation of Nrf2 Signaling Pathway

**Figure 4.**
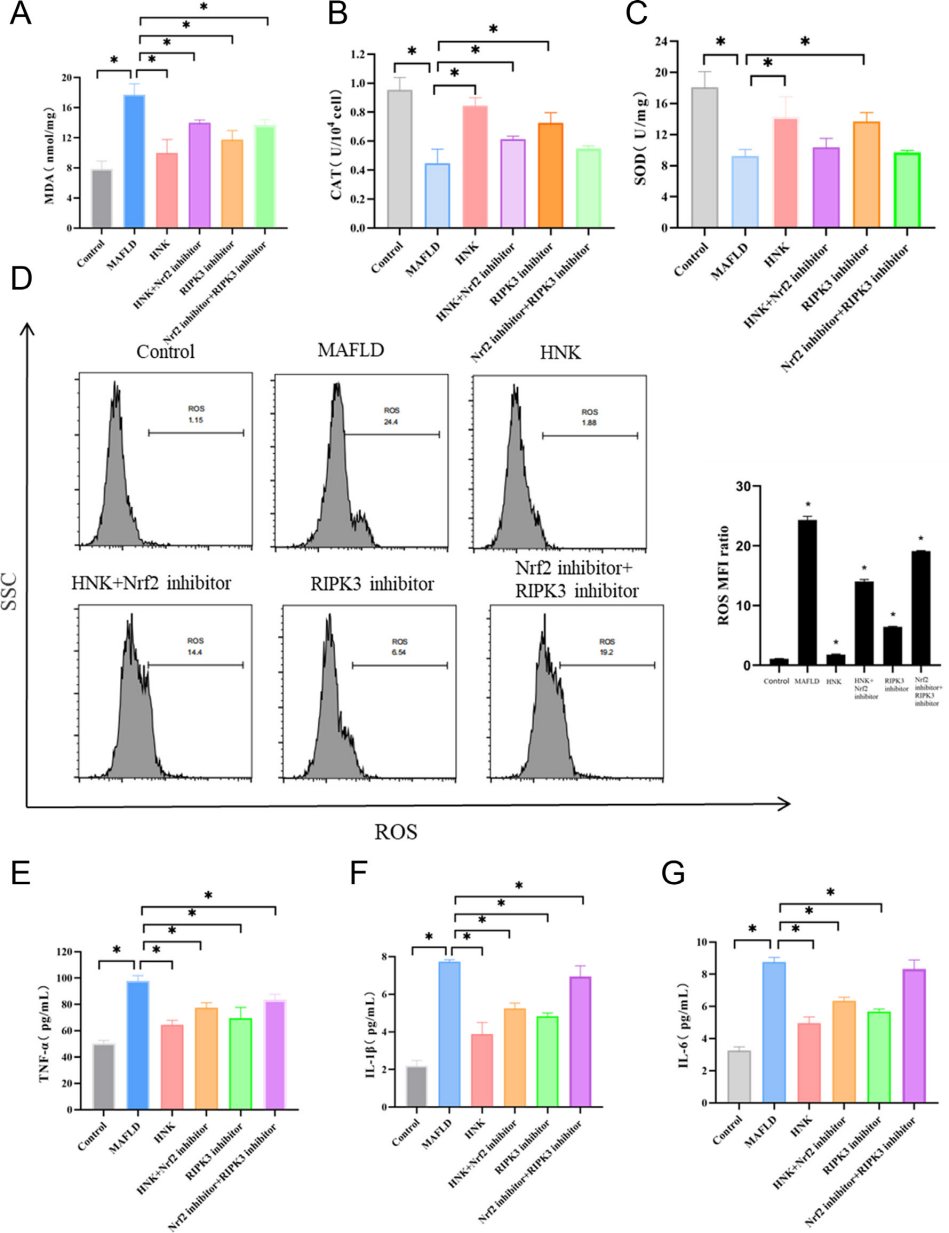
HNK inhibited MAFLD progression by activating the Nrf2 signaling pathway mediated antioxidant activity. (A-C) The concentrations of MDA (A), activity of CAT (B), and SOD (C) in LO2 cells with different treatments were determined by the biochemistry detection kits. (D) Flow cytometry was used to detect the ROS level in LO2 cells with different treatments. (E-G) The concentrations of TNF-α (E), IL-1β (F), and IL-6 (G) (pm/mL) in LO2 cells with different treatments. n = 3, *P < .05 between 2 groups. Student’s *t*-test was used to compare differences between the 2 groups.

Thereafter, the antioxidation and anti-inflammation functions of HNK on MAFLD were verified. On the one hand, the concentration of MDA and ROS ratio showed a significant increase in the MAFLD group, which was reduced by HNK treatment. With the treatment of an Nrf2 inhibitor or RIPK3 inhibitor, the concentrations of MDA and ROS ratio were increased compared to the HNK group (*P* < .05) (Figure 4A and 4D). On the other hand, the activities of CAT and SOD were significantly decreased in the MAFLD group and enhanced by HNK. With the treatment of an Nrf2 inhibitor or RIPK3 inhibitor, the activities of CAT and SOD were decreased compared to the HNK group (*P* < .05) (Figure 4B and 4C). Furthermore, the concentrations of inflammatory factors (TNF-α, IL-1β, and IL-6) were highest in the MAFLD group while lowest in the HNK group. The concentrations of inflammatory factors were increased with the treatment of an Nrf2 inhibitor or RIPK3 inhibitor, especially with the treatment of both (*P* < .05) (Figure 4E-G). These findings reveal that HNK suppressesMAFLD by antioxidation and antiinflammation effect via the activation of the Nrf2 signaling pathway.

## Discussion

Metabolic-associated fatty liver disease is a common chronic liver disease worldwide, mainly characterized by excessive lipid accumulation in the liver.^2,21^ Metabolic-associated fatty liver disease leads to oxidative stress response, massive inflammatory lesions, reactive fibrosis, and necrotic feedback in liver cells.^22^ With the continuous improvement in people’s quality of life, the number of patients with MAFLD is gradually increasing, and the prevention and treatment of the disease are gradually attracting more attention.^23^ However, there is currently a lack of effective drugs for the treatment of MAFLD. Here, our results showed that HNK inhibited MAFLD by activating the Nrf2 signaling pathway to play an antioxidant and antiinflammatory role.

Oxidative stress is widely recognized to be vital in the occurrence and development of MAFLD.^16^ During the progression of MAFLD, excessive lipid deposition can provide a reaction matrix for lipid peroxidation damage, leading to the accumulation of lipid peroxidation products and causing oxidative stress and lipid peroxidation in liver cells, eventually leading to the further deterioration of MAFLD.^24^ Nrf2 is one of the most necessary endogenous antioxidant stress pathways in cells.^25^ Several studies have shown that many drugs can mitigate symptoms of the diseases via the Nrf2 pathway. For example, baicalin exerts neuroprotective actions by regulating the Nrf2–NLRP3 axis.^26^ Under normal conditions, Nrf2 is in the cytoplasm and binds to the specificity receptor Kelch-like epoxide propane-associated protein-1 (Keap1) in the form of dimerization.^27^ Under oxidative stress and other conditions, Nrf2 dissociates from Keap1 and translocates into the nucleus.^28^ By binding with antioxidant response elements, it initiates the expression of antioxidant enzyme genes such as quinone oxidoreductase-1 (NQO1) and heme oxygenase-1 (HO-1), inhibits the release of ROS, and ultimately exerts antioxidant capacity.^29^ The antioxidant mediated by Nrf2 plays a protective role in the progression of MAFLD.^30^ Wang et al showed that dietary selenium supplementation could alleviate obesity-induced oxidative stress and MAFLD in mouse livers by regulating the Keap1/Nrf2 pathway.^31^ In addition, a study showed that HNK attenuated hepatic oxidative damage and insulin resistance by upregulating the expression of Nrf2.^32^ In our study, the results indicated the HNK suppressed oxidative stress in MAFLD in vitro, and with the treatment of an Nrf2 inhibitor, protein expression levels of Nrf2, HO-1, and NQOI were significantly decreased compared with the HNK group. Therefore, we speculate that HNK may inhibit the progression of MAFLD by mediating antioxidant activity through the activation of the Nrf2 signaling pathway.

A previous research has shown that activation of the Nrf2 signaling pathway can prevent ethanol-induced liver cell necroptosis.^33^ Necroptosis is a caspase-independent programmed cell death, in which mixed MLKL is recruited to form the necrosome complex composed of RIPK1 and RIPK3 in response to tumor necrosis factor.^34^ Vinpocetine mitigates methotrexate-induced duodenal intoxication by modulating RIPK1/RIPK3/MLKL signals.^35^ Recently, a study indicated that necroptosis is necessary for the progression of MAFLD.^36^ Afonso et al^17^ found that the expression level of RIPK3 was increased in MAFLD patients and its knockout could significantly inhibit mouse liver inflammation and carcinogenesis. Hao et al^37^ demonstrated that berberine could alleviate the progression of mouse MAFLD by activating the Nrf2 pathway, accompanied by downregulation of RIPK3 expression, suggesting that Nrf2 may be associated with RIPK3-mediated necroptosis in the development of MAFLD. In addition, Zhang et al^18^ found that intermittent hypoxia could exacerbate MAFLD by promoting RIPK3-dependent necroptosis and inhibiting the Nrf2 signaling pathway. In this study, with the treatment of RIPK3 inhibitor, the lipid droplet was decreased compared with the MAFLD group, while cell survival was increased. The antioxidation and antiinflammation effect of HNK on MAFLD was lessened by the RIPK3 inhibitor. Therefore, it suggested that the Nrf2 pathway and RIPK3-dependent necroptosis may form a negative feedback axis and participate importantly in the progression of MAFLD. HNK suppressed the MAFLD by antioxidation and antiinflammation effect via the activation of the Nrf2 signaling pathway.

Herein, we investigated the protective role of HNK by the MAFLD cell model. The potential therapeutic effect of HNK on MAFLD features, including inflammation and oxidative stress, was evaluated. Nevertheless, some limits were obvious in this study, for instance, the protective role of HNK in MAFLD should be verified in vivo. The underlying mechanism of the Nrf2 pathway and RIPK3-dependent necroptosis needs more investigation.

In conclusion, the study indicates that HNK suppresses oxidative stress and inflammation in MAFLD via activation of the Nrf2 signaling pathway. The Nrf2 pathway and RIPK3-dependent necroptosis may form a negative feedback axis and participate importantly in the progression of MAFLD. This study provides a potential treatment compound for MAFLD.

## Figures and Tables

**Supplementary Table 1.** The Data of Figure 1B

	Control	MAFLD	HNK low	HNK middle	HNK high
1	1.07	24.7	11.2	1.18	4.49
2	1.01	24.5	10.7	1.19	4.87
3	1.04	24.3	11.6	1.34	4.72

HNK, honokiol; MAFLD, metabolic-associated fatty liver disease.

**Supplementary Table 2.** The Data of Figure 1C

	Control	MAFLD	HNK low	HNK middle	HNK high
1	98.4505	35.4817	57.669	90.815181	74.9158
2	102.403	33.0564	50.4828	103.30115	77.9699
3	100.247	37.0986	54.0759	97.552212	72.221
4	94.1388	36.2003	54.9742	95.845497	71.5024
5	99.2589	33.8648	62.34	99.977543	77.2513
6	101.684	35.1224	47.3389	101.5046	68.1788
7	104.289	28.3854	54.3454	104.82821	75.8141
8	99.5284	32.8767	53.7166	99.887716	71.2329

HNK, honokiol; MAFLD, metabolic-associated fatty liver disease.

**Supplementary Table 3.** The Data of Figure 1D

	Control	MAFLD	HNK low	HNK middle	HNK high
1	8.66722	14.5455	14.1912	10.655172	12.2027
2	7.97129	15.8713	12.8534	9.1138153	11.0122
3	8.41148	16.4196	12.8727	10.024014	11.7125

HNK, honokiol; MAFLD, metabolic-associated fatty liver disease.

**Supplementary Table 4.** The Data of Figure 1E

	Control	MAFLD	HNK low	HNK middle	HNK high
1	16.974	10.0744	11.4243	14.433157	13.9519
2	17.0705	8.37383	13.0345	15.922465	12.1495
3	16.0386	10.0344	13.7072	14.241211	15.5123

HNK, honokiol; MAFLD, metabolic-associated fatty liver disease.

**Supplementary Table 5.** The Data of Figure 1F

	Ethics Committee Approval	MAFLD	**HNK Low**	HNK middle	HNK high
1	0.86648	0.37154	0.73088	0.744444	0.61156
2	0.9275	0.43392	0.48952	0.679356	0.60884
3	0.79462	0.40002	0.48952	0.734952	0.63868

HNK, honokiol; MAFLD, metabolic-associated fatty liver disease.

**Supplementary Table 6.** The 2-△△Ct Value of The Genes in Figure 2A

	Control			MAFLD			HNK			HNK+Nrf2 inhibitor			RIPK3 inhibitor			Nrf2 inhibitor+ RIPK3 inhibitor		
TGF-β1	1.1	1	1	1.9	1.78	1.728	1.11	1.02	1.1	1.54	1.6	1.6	1.43	1.37	1.4	1.7	1.7	1.6
TIMP1	1	1.1	1	2.1	2.12	1.981	1.15	1.04	1.2	1.61	1.5	1.5	1.38	1.43	1.4	1.88	1.9	1.8
Collagen I	1	1	1	1.8	1.88	1.791	1.14	1.13	1.2	1.44	1.5	1.5	1.24	1.3	1.3	1.53	1.6	1.5

HNK, honokiol; MAFLD, metabolic-associated fatty liver disease.

**Supplementary Table 7.** The Relative Expression of The Proteins in Figure 2

	Control			MAFLD			HNK			HNK+Nrf2 inhibitor			RIPK3 inhibitor			Nrf2 inhibitor+ RIPK3 inhibitor		
TGF-β1	0.4	0.3	0	1.4	1.63	1.564	0.45	0.5	0.5	0.87	0.9	0.9	0.63	0.75	0.7	1.18	1.2	1.1
TIMP1	0.3	0.3	0	1.4	1.56	1.617	0.47	0.47	0.5	0.9	0.9	0.6	0.64	0.71	0.67	1.26	1.3	1.1
Collagen I	0.3	0.3	0	1.5	1.79	1.776	0.66	0.71	0.7	1.23	1.3	1.1	0.95	1.02	0.98	1.57	1.5	1.5
Nrf2	1.7	1.6	2	0.3	0.33	0.306	1.35	1.33	1.4	0.82	0.8	1	1.07	1.17	1.06	0.61	0.5	0.5
HO-1	1.6	1.6	2	0.3	0.22	0.327	1.19	1.05	1.2	1.05	0.7	0.9	1.01	0.84	1.16	0.59	0.6	0.8
NQO1	1.8	1.6	2	0.5	0.49	0.547	1.34	1.2	1.1	1.11	1	0.9	1.1	1.07	1.06	0.8	0.6	0.7
RIP1	0.3	0.3	0	1.7	1.81	1.639	0.57	0.56	0.5	1.28	1.2	1	0.96	0.96	0.95	1.65	1.5	1.3
RIP3	0.3	0.3	0	1.5	1.68	1.691	0.57	0.64	0.5	1.05	1.1	1	0.78	1.04	0.84	1.42	1.5	1.2
MLKL	0.4	0.4	0	1.5	1.74	1.831	0.7	0.67	0.7	1.17	1.3	1.1	1.02	1.04	0.92	1.6	1.5	1.3

HNK, honokiol; MAFLD, metabolic-associated fatty liver disease.

**Supplementary Table 8.** The Data of Figure 3B

	Control	MAFLD	HNK	HNK+Nrf2 inhibitor	RIPK3 inhibitor	Nrf2 inhibitor+ RIPK3 inhibitor
1	102.23	31.6572	71.7305	53.19949812	67.02055786	37.67976064
2	96.4386	26.87	72.3482	47.94903967	60.38027217	42.0036676
3	104.546	28.8775	62.6194	53.04507287	59.37650806	33.51027893
4	95.9753	30.5762	76.9038	45.86429881	68.33317247	37.52533539
5	98.9094	28.8775	76.981	48.10346492	67.7926841	37.67976064
6	105.472	29.2636	71.2673	44.39725895	65.16745488	34.12797992
7	95.5892	26.0979	69.1053	43.77955796	58.2183187	33.7419168
8	100.84	25.1713	64.0093	49.10722903	63.70041502	41.77202973

HNK, honokiol; MAFLD, metabolic-associated fatty liver disease.

**Supplementary Table 9.** The Data of Figure 4A

	Control	MAFLD	HNK	HNK+Nrf2 inhibitor	RIPK3 inhibitor	Nrf2 inhibitor+ RIPK3 inhibitor
1	6.99	16.24	10.36	13.79	13.07	14.39
2	7.46	17.78	8.02	14.44	10.72	12.91
3	9.03	19.14	11.55	13.76	11.42	13.73

HNK, honokiol; MAFLD, metabolic-associated fatty liver disease.

**Supplementary Table 10.** The Data of Figure 4B

	Control	MAFLD	HNK	HNK+Nrf2 inhibitor	RIPK3 inhibitor	Nrf2 inhibitor+ RIPK3 inhibitor
1	20.21	8.577	17.215	11.526	13.332	9.586
2	17.83	8.896	13.286	10.321	12.714	10.015
3	16.16	10.199	12.183	9.176	14.967	9.605

HNK, honokiol; MAFLD, metabolic-associated fatty liver disease.

**Supplementary Table 11.** The Data of Figure 4C

	Control	MAFLD	HNK	HNK+Nrf2 inhibitor	RIPK3 inhibitor	Nrf2 inhibitor+ RIPK3 inhibitor
1	1.04	0.367	0.850	0.594	0.704	0.563
2	0.87	0.556	0.895	0.606	0.805	0.53
3	0.95	0.419	0.780	0.637	0.663	0.555

HNK, honokiol; MAFLD, metabolic-associated fatty liver disease.

**Supplementary Table 12.** The Data of Figure 4D

	Control	MAFLD	HNK	HNK+Nrf2 inhibitor	RIPK3 inhibitor	Nrf2 inhibitor+ RIPK3 inhibitor
1	1.15	24.4	1.880	14.4	6.54	19.2
2	1.01	23.7	1.700	13.8	6.36	19
3	1.05	24.9	1.790	13.9	6.42	19.1

HNK, honokiol; MAFLD, metabolic-associated fatty liver disease.

**Supplementary Table 13.** The Data of Inflammatory Factors in Figure 4E-G

	Control			MAFLD			HNK			HNK+Nrf2 inhibitor			RIPK3 inhibitor			Nrf2 inhibitor+ RIPK3 inhibitor		
TNF-α	48.7	53.018	49.318	102.334	96.548	93.983	67.344	60.466	65.464	73.635	77.426	81.229	76.793	60.466	71.114	86.316	78.692	85.679
IL-1β	1.909	2.477	2.208	7.687	7.656	7.845	4.043	3.197	4.407	5.508	4.956	5.293	4.895	4.62	4.956	7.467	7.028	6.342
IL-6	3.161	3.06304	3.51481	8.452666	8.79446	9.025563	5.30041	4.52561	5.02628	6.59349	6.29202	6.15998	5.80309	5.48662	5.73919	8.8328	8.41505	7.71413

HNK, honokiol; MAFLD, metabolic-associated fatty liver disease.
